# Increased Local Inflammatory Response to MOC31PE Immunotoxin After Cytoreductive Surgery and Hyperthermic Intraperitoneal Chemotherapy

**DOI:** 10.1245/s10434-021-10022-0

**Published:** 2021-05-21

**Authors:** Ebbe Billmann Thorgersen, Jørund Asvall, Ida Storhaug Frøysnes, Camilla Schjalm, Stein Gunnar Larsen, Svein Dueland, Yvonne Andersson, Øystein Fodstad, Tom Eirik Mollnes, Kjersti Flatmark

**Affiliations:** 1 Department of Gastroenterological Surgery, Oslo University Hospital The Radium Hospital, Oslo, Norway; 2 Department of Immunology, Oslo University Hospital Rikshospitalet, Oslo, Norway; 3 Division of Emergencies and Critical Care, Oslo University Hospital, Oslo, Norway; 4 Institute of Clinical Medicine, University of Oslo, Oslo, Norway; 5 Department of Tumor Biology, Oslo University Hospital The Radium Hospital, Oslo, Norway; 6 Faculty of Medicine, University of Oslo, Oslo, Norway; 7 Department of Oncology, Oslo University Hospital The Radium Hospital, Oslo, Norway; 8 Research Laboratory, Nordland Hospital, Bodø, and Faculty of Health Sciences, K.G. Jebsen TREC, University of Tromsø, Tromsø, Norway; 9 Centre of Molecular Inflammation Research, Norwegian University of Science and Technology, Trondheim, Norway

## Abstract

**Background:**

Despite extensive cytoreductive surgery and hyperthermic intraperitoneal chemotherapy (CRS-HIPEC), most patients with resectable peritoneal metastases from colorectal cancer experience disease relapse. MOC31PE immunotoxin is being explored as a novel treatment option for these patients. MOC31PE targets the cancer-associated epithelial cell adhesion molecule, and kills cancer cells by distinct mechanisms, simultaneously causing immune activation by induction of immunogenic cell death (ICD).

**Methods:**

Systemic and local cytokine responses were analyzed in serum and intraperitoneal fluid samples collected the first three postoperative days from clinically comparable patients undergoing CRS-HIPEC with (*n* = 12) or without (*n* = 26) intraperitoneal instillation of MOC31PE. A broad panel of 27 pro- and antiinflammatory interleukins, chemokines, interferons, and growth factors was analyzed using multiplex technology.

**Results:**

The time course and magnitude of the systemic and local postoperative cytokine response after CRS-HIPEC were highly compartmentalized, with modest systemic responses contrasting substantial intraperitoneal responses. Administration of MOC31PE resulted in changes that were broader and of higher magnitude compared with CRS-HIPEC alone. Significantly increased levels of innate proinflammatory cytokines, such as interleukin (IL)-6, IL-1β, and tumor necrosis factor (TNF) as well as an interesting time response curve for the strong T-cell stimulator interferon (IFN)-γ and its associated chemokine interferon gamma-induced protein/chemokine (C-X-C motif) ligand 10 (IP-10) were detected, all associated with ICD.

**Conclusions:**

Our study revealed a predominately local rather than systemic inflammatory response to CRS-HIPEC, which was strongly enhanced by MOC31PE treatment. The MOC31PE-induced intraperitoneal inflammatory reaction could contribute to improve remnant cancer cell killing, but the mechanisms remain to be elucidated in future studies.

**Supplementary Information:**

The online version contains supplementary material available at 10.1245/s10434-021-10022-0.

Cytoreductive surgery (CRS) and hyperthermic intraperitoneal chemotherapy (HIPEC) is established as standard-of-care treatment for peritoneal metastasis from colorectal cancer (PM-CRC) in selected patients with resectable disease and for pseudomyxoma peritonei (PMP).^1^^,^^2^ CRS-HIPEC involves potentially extensive procedures to ensure resection of all visible tumor tissue within the peritoneal cavity, with subsequent perfusion of the peritoneal cavity with heated chemotherapy.^3^ Although CRS-HIPEC may improve overall survival (OS), the majority of patients experience disease relapse, and novel treatment options are therefore needed.^4^

MOC31PE immunotoxin consists of the MOC31 monoclonal antibody covalently coupled to *Pseudomonas* exotoxin (PE). MOC31PE binds to the tumor-associated EpCAM on the surface of cancer cells, through which it is internalized, and PE is released to trigger rapid cell death.^5^^,^^6^ With EpCAM being ubiquitously expressed in most epithelial cancers, MOC31PE is being developed as a promising new anticancer drug.^7^^,^^8^ In the recent phase I/II ImmunoPeCa trial, MOC31PE administered intraperitoneally the day after CRS-HIPEC was well tolerated, with excellent drug stability and pharmacokinetic properties and with promising long-term results.^9^^,^^10^

In addition to direct cytotoxic effects, we recently showed that MOC31PE induced immune-modulating effects, suggesting that tumor cells undergo immunogenic cell death (ICD).^8^^,^^11^ In the context of MOC31PE being administered intraperitoneally after surgery, tumor-related ICD could be reinforced by endogenous factors upon release of damage-associated molecular patterns (DAMPs) from cells in the peritoneal cavity.^12^ DAMPs, e.g., calreticulin and high-mobility group protein B1 (HMGB1),^13^^,^^14^ are released after comprehensive surgery with subsequent activation of the inflammatory cascade through Toll-like receptor 4 and its coreceptor CD14,^15^^,^^16^ subsequently inducing production and release of a range of cytokines, such as TNF, IL-1β, IL-6, IL-8, and IL-10.^17^^,^^18^ Potentiation of MOC31PE’s effect by endogenous DAMPs could therefore contribute to the magnitude of inflammation and potential antitumor effects.

Thus, the aim of the present study is to explore these potential effects in the context of intraperitoneal treatment of PM-CRC by analyzing the local and systemic cytokine responses in patients undergoing CRS-HIPEC with or without additional MOC31PE treatment.

## Patients and Methods

### Patients and Treatments

The ImmunoPeCa trial included 21 adult patients with suspected PM from histologically verified EpCAM-positive CRC selected for CRS-HIPEC. All patients underwent treatment with complete CRS and standard mitomycin C (MMC)-based HIPEC (35 mg/m^2^, maximum 70 mg), administered for 90 min in three fractions at 41.5 °C using a “closed technique with open abdomen.” Briefly, after CRS (“open abdomen”) and before the HIPEC procedure, a frame and a plastic wrap was mounted and adapted to the surgical field (“closed technique”) as a measure to protect operating theater personnel from chemotherapy exposure. Patients were given MOC31PE as single rapid intraperitoneal instillations through two abdominal drainage catheters the morning after CRS-HIPEC. The catheters were clamped for 6 h after drug instillation, then reopened to remove excess intraabdominal fluid. Standard dose escalation was applied (3 + 3 design), with four dose levels (2.5, 5.0, 7.5, and 10 µg/kg). The fourth dose level (10 µg/kg) included 12 patients, who are referred to as the MOC31PE group. For additional study details, see Froysnes et al.^9^ The Accordion Severity Grading System of Surgical Complications was used to compare surgical complications between the groups (Table 1).^19^ The CRS-HIPEC group was recruited from patients treated with MMC-based CRS-HIPEC for PM-CRC or PMP in our institution. Of 30 patients included, 4 were deemed unresectable because of high tumor burden at time of surgery, did not receive CRS-HIPEC, and were excluded from the study. Hence, 26 patients were included in the CRS-HIPEC group.

**Table 1:** Patient characteristics

	CRS-HIPEC + MOC31PE (*n* = 12)	CRS-HIPEC (*n* = 26)	Significance level (*P*-value)^a^
Gender			1.0
Female	10 (83)^b^	20 (77)	
Male	2 (17)	6 (23)	
ASA^c^			0.768
ASA 1–2	11 (92)	23 (88)	
ASA 3–4	1 (8)	3 (12)	
ECOG^d^			0.408
ECOG 0	10 (83)	24 (92)	
ECOG 1	2 (17)	2 (8)	
Age (years)	53 (29–73)^e^	62 (37–76)	0.376
BMI^f^	25 (22.9–29.7)	26.2 (20–40.7)	0.745
Diagnosis			0.018
PMP^g^	–	8 (31)	
PM-CRC^h^	12 (100)	17 (65)	
Other	–	1 (4)	
Origin			0.191
Appendix	1 (8)	10 (38)	
Ascendens	2 (17)	6 (23)	
Transversum	1 (8)	2 (8)	
Descendens	2 (17)	1 (4)	
Rectosigmoid	6 (50)	6 (23)	
Small bowel	0 (0)	1 (4)	
CEA	3.2 (1.2–12)	3.3 (0.8–55)	0.653
CA 19-9	10 (5–92)	13.5 (5–764)	0.327
CA 125	20 (7–42)	15 (4–84)	0.441
Operating time (min)	465 (304–685)	420 (270–832)	0.207
Operative blood loss (mL)	500 (100–1100)	300 (100–1500)	0.057
Peroperative transfusion			
RBC (units)	0 (0–2)	0 (0–2)	0.676
Plasma (units)	0 (0–2)	0 (0–4)	0.841
Platelets (units)	–	–	–
Peritoneal cancer index (PCI)	7 (0–20)	5 (0–31)	0.328
Completeness of cytoreduction (CC)			0.497
CC 0	12 (100)	25 (96)	
CC 1	0 (0)	1 (4)	
Length of stay (days)	10 (8–18)	9 (4–20)	0.566
Accordion^i^			0.339
3	1 (8)	1 (4)	
4	–	1 (4)	
CRP screening	4.9 (1–45)	1.7 (1–106)	0.238

^a^Mann–Whitney *U* test, except gender (Fisher exact test), diagnosis, and origin (likelihood ratio)

^b^Number of patients and percentage

^c^American Society of Anesthesiologists physical status classification system

^d^Eastern Cooperative Oncology Group performance status

^e^Median and range

^f^Body mass index

^g^Pseudomyxoma peritonei

^h^Peritoneal metastasis (from) colorectal cancer

^i^Accordion Severity Grading System of Surgical Complications ≥ 3

### Sampling and Processing

Blood samples were collected at baseline (prior to CRS-HIPEC) and daily postoperatively at standardized time points, processed to serum by centrifugation at 2500×*g* for 10 min, then stored at − 80 °C until analysis. Peritoneal fluid was collected twice daily for the first three postoperative days from either of the indwelling abdominal drains and stored at − 80 °C until analysis. The samples were centrifuged at 3000×*g* for 15 min after thawing.

### Total Protein Measurement

The protein concentration in serum samples and peritoneal fluid was measured by Bio-Rad Protein Assay (Bio-Rad Laboratories, Hercules, CA, USA) and Bio-Rad Protein Assay Standard II (Bio-Rad Laboratories, Hercules, CA, USA).

### Cytokine Analysis

Serum and peritoneal fluid samples were thawed on crushed ice and analyzed using a multiplex cytokine assay (Bio-Plex Human Cytokine 27-Plex Panel; Bio-Rad Laboratories Inc., Hercules, CA, USA) containing the following cytokines, chemokines, and growth factors: IL-1β, IL-1 receptor antagonist (IL-1Ra), IL-2, IL-4, IL-5, IL-6, IL-7, IL-8 (CXCL8), IL-9, IL-10, IL-12 p70, IL-13, IL-15, IL-17, eotaxin (CCL11), basic fibroblast growth factor (bFGF), granulocyte colony stimulating factor (G-CSF), granulocyte-macrophage colony stimulating factor (GM-CSF), IFN-γ, IP-10 (CXCL10), monocyte chemotactic protein 1 (MCP-1, or CCL2), macrophage inflammatory protein-1α (MIP-1α, or CCL3), macrophage inflammatory protein-1β (MIP-1β, or CCL4), platelet-derived growth factor-BB (PDGF-BB), regulated upon activation T cell expressed and secreted (RANTES, or CCL5), TNF, and vascular endothelial growth factor (VEGF). The analysis was performed according to manufacturer instructions.

### Bio-Plex Standard Curve Validation

As the multiplex kit is developed for plasma and serum samples and the exact matrix in peritoneal fluid is not known, we tested whether the standard curve of each cytokine was affected by different matrixes. The standard curve with the kit’s standard diluent was compared with standard curves with the standard diluent added 0.5% bovine serum albumin (BSA) or 25% Voluven (containing 60 mg/mL hydroxyethyl starch) (Fresenius Kabi Deutschland GmbH, Bad Homburg v.d.H., Germany).

### Data Presentation and Statistical Analysis

Clinicopathological data are presented as median and range or percentage of the study population. Mann–Whitney *U* test, Fisher exact test, or likelihood ratio was used depending on data characteristics. Cytokine data were assumed not to be normally distributed and are presented as box-and-whiskers plot (Turkey). Individual areas under the curve (AUC) were calculated for all cytokines, with comparisons between the MOC31PE and CRS-HIPEC groups using the Mann–Whitney *U* test. GraphPad Prism 7.02 (GraphPad Software, San Diego, CA, USA) and SPSS software (version 25, IBM SPSS, Chicago, IL, USA) were used for the analysis.

### Ethics

The ImmunoPeCa trial (NCT00769405) was conducted in accordance with the International Conference on Harmonization Good Clinical Practice guidelines and was approved by the Norwegian Medicines Agency, the Regional Ethics Committee, and local health authorities. An amendment to the project Peritoneal Surface Malignancies—Characterization, Models and Treatment Strategies (NCT02073500), to include 30 patients in the CRS-HIPEC group, was approved by the Regional Ethics Committee and approved by the Data Protection Official for Research for Oslo University Hospital. All patients gave written informed consent before study entry.

## Results

### Patient Characteristics

More females than males were included in the study, with 83% in the MOC31PE group and 77% in the CRS-HIPEC group (Table 1). A range of clinicopathological parameters were compared, and there were no significant differences between the groups (Table 1). The median Peritoneal Cancer Index (PCI) was 7 with a range of 0–20 in the MOC31PE group and 5 in the CRS-HIPEC group with a range of 0–31. Only patients with PM-CRC were included in the MOC31PE group, while 8 patients (31%) with PMP were included in the CRS-HIPEC group, 17 patients (65%) had PM-CRC, and 1 patient included had PM from a small bowel adenocarcinoma.

### Total Protein Measurements

The total protein measurement displayed no significant differences in the MOC31PE group compared with the CRS-HIPEC group in peritoneal fluid and serum (Supplementary Fig. S1). These results indicate that the amount of protein in the samples did not influence the results.

### Standard Curve Validation

The standard curves with standard diluent added 0.5% BSA and 25% Voluven had exactly the same configuration as the standard curve with the standard diluent as the kit (Supplementary Fig. S2). Thus, standard buffers were used for analyses.

### Serum Cytokine Analysis

In general, serum values were 2- to 1000-fold lower than the corresponding intraperitoneal values, and eight of the cytokines (IL-5, IL-10, IL-12, IL-13, IL-15, G-CSF, GM-CSF, and VEGF) were below the detection limit at all time points in serum. However, some cytokines showed the same response pattern as in peritoneal fluid, e.g., IL-6, with a peak value on the first postoperative day followed by a gradual decrease. The MOC31PE and CRS-HIPEC groups exhibited relatively similar profiles, and some of the differences observed were difficult to interpret because of low overall values (such as for IL-1β). Interestingly, IP-10 showed a similar time-dependent significant increase in the MOC31PE group compared with the CRS-HIPEC group as was seen in peritoneal fluid. For details regarding the serum findings, see Supplementary Data and Supplementary Figs. S3–7.

### Peritoneal Fluid Sample Cytokine Analysis

In general, all 27 cytokines measured were detected in peritoneal fluid, whereas in serum eight were not detectable. Overall, the MOC31PE group recorded significantly higher levels than the CRS-HIPEC group, with some variations between the cytokines as described below. The general pattern of response could be divided into three. One was an immediate rise postoperatively in both groups and a subsequent gradual decline, e.g., for IL-6 and IL-10. For some cytokines, this rise was more pronounced in the MOC31PE group, e.g., IL-1β and G-CSF. An almost flat time-dependent course was observed in both groups for other cytokines, e.g., TNF and IL-7. Finally, for a few cytokines, a time-dependent increase, in particular in the MOC31PE group, could be demonstrated, e.g., for MCP-1, IFN-γ, and IP-10.

### Proinflammatory Cytokines

**Fig. 1:**
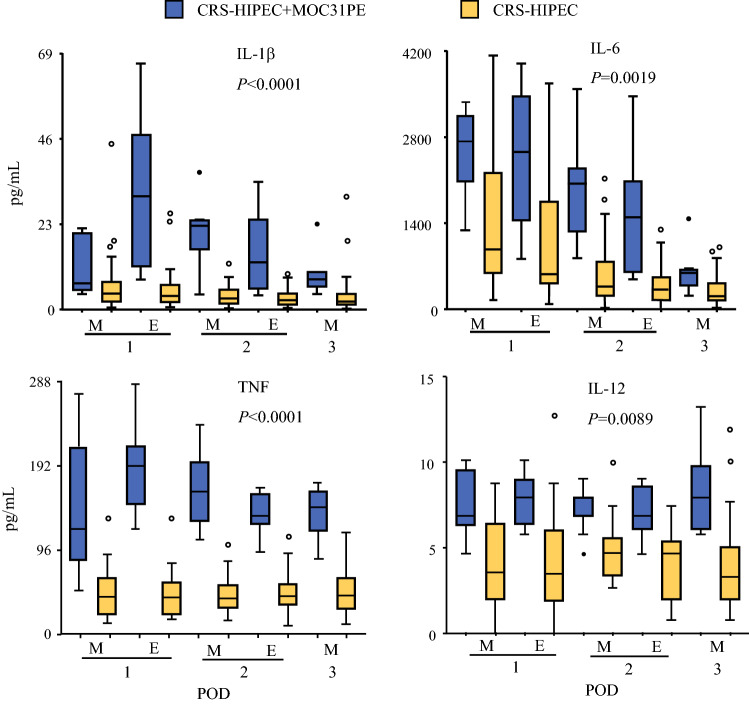
Proinflammatory cytokine responses in peritoneal fluid. Analysis of proinflammatory cytokines IL-1β, IL-6, TNF, and IL-12 in peritoneal fluid samples on the first three postoperative days after CRS-HIPEC with (*n* = 12, blue boxes) or without (*n* = 26, yellow boxes) intraperitoneal instillation of MOC31PE immunotoxin. Data presented as median and box-and-whiskers plot with outliers. *POD* postoperative day (1–3), *M* morning sample, *E* evening sample. *P*-values < 0.05 considered statistically significant

The measured levels of proinflammatory cytokines were significantly higher in the MOC31PE group than in the CRS-HIPEC group, but exhibited time-dependent variability (Fig. 1). IL-1β showed a time-dependent increase and sequential decrease the first three days postoperatively in both groups. IL-6 showed a time-dependent decrease the first three days postoperatively in both groups. TNF showed a time-dependent increase then a slight decrease the first three days postoperatively in the MOC31PE but not the CRS-HIPEC group, while for IL-12, no major time-dependent change was detected in either group.

### Chemokines

**Fig. 2:**
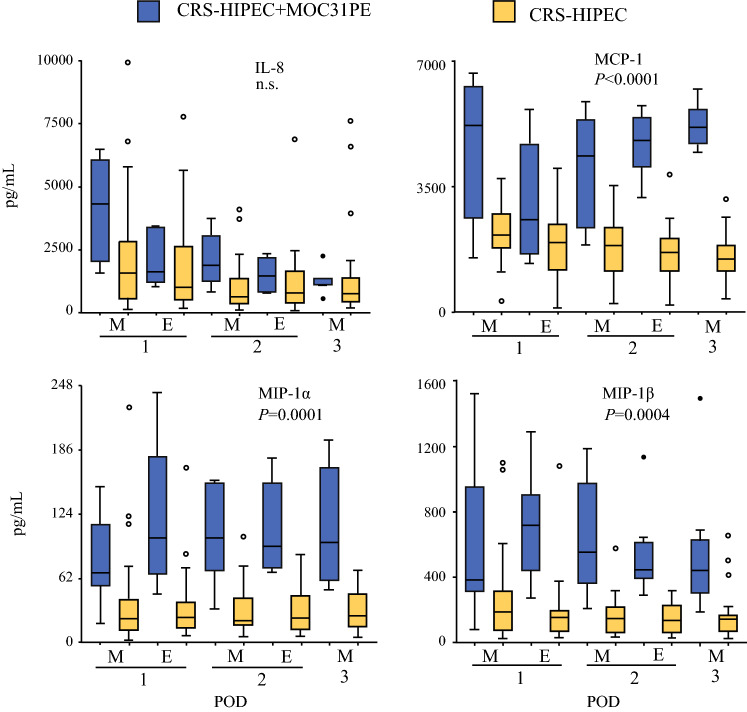
Chemokine responses in peritoneal fluid. Analysis of chemokines IL-8, MCP-1, MIP-1α, and MIP-1β in peritoneal fluid samples on the first three postoperative days after CRS-HIPEC with (*n* = 12, blue boxes) or without (*n* = 26, yellow boxes) intraperitoneal instillation of MOC31PE immunotoxin. Data presented as median and box-and-whiskers plot with outliers. *POD* postoperative day (1–3), *M* morning sample, *E* evening sample. *P*-values < 0.05 considered statistically significant. *n.s.* nonsignificant

IL-8 showed a time-dependent decrease the first three days postoperatively in both groups (Fig. 2). There was no significant difference between the two groups, but the values were higher for the MOC31PE than CRS-HIPEC group at two intercepted time points. MCP-1 showed a time-dependent increase in the MOC31PE group compared with the CRS-HIPEC group from the morning sample on the second postoperative day (M 2) and onwards. For both MIPs, the level in the MOC31PE group was significantly higher compared with the CRS-HIPEC group.

### Growth Factors

**Fig. 3:**
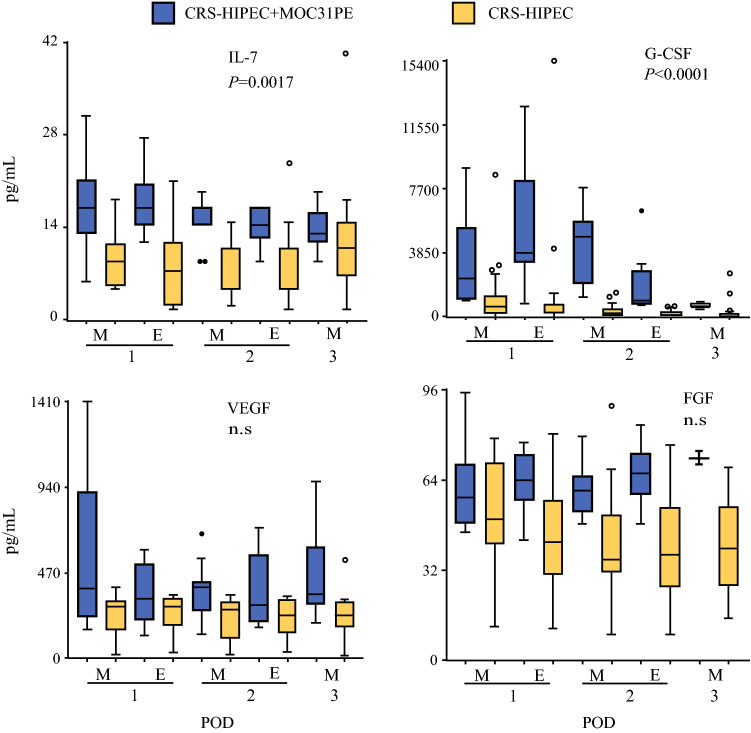
Growth factor responses in peritoneal fluid. Analysis of growth factors G-CSF, VEGF, IL-7, and FGF in peritoneal fluid samples on the first three postoperative days after CRS-HIPEC with (*n* = 12, blue boxes) or without (*n* = 26, yellow boxes) intraperitoneal instillation of MOC31PE immunotoxin. Data presented as median and box-and-whiskers plot with outliers. *POD* postoperative day (1–3), *M* morning sample, *E* evening sample. *P*-values < 0.05 considered statistically significant. *n.s*. nonsignificant

For IL-7, no major time-dependent change was detected in either group (Fig. 3). The values were significantly higher in the MOC31PE than CRS-HIPEC group. G-CSF showed a time-dependent increase and sequential decrease in the MOC31PE group the first three days postoperatively, while in the CRS-HIPEC group, a time-dependent decrease was observed from the first time point and onwards. The values were significantly higher in the MOC31PE than CRS-HIPEC group. No major time-dependent change in VEGF levels was detected in either group. There was no significant difference between the two groups. For FGF, the MOC31PE group showed a time-dependent increase from the evening sample of the first postoperative day (E1) compared with the CRS-HIPEC group, who exhibited a decrease over time from the first time point.

### IFN-γ, IP-10, and Antiinflammatory Cytokines IL-1RA and IL-10

**Fig. 4:**
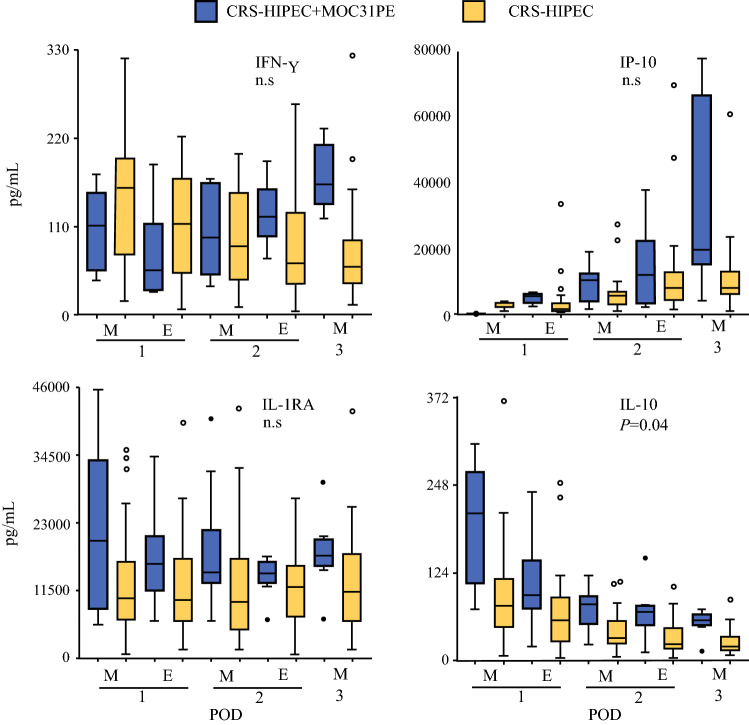
IFN-γ, IP-10, IL-1RA, and IL-10 responses in peritoneal fluid. Analysis of IFN-γ and IP-10, RANTES, and antiinflammatory cytokine IL-10 in peritoneal fluid samples on the first three postoperative days after CRS-HIPEC with (*n* = 12, blue boxes) or without (*n* = 26, yellow boxes) intraperitoneal instillation of MOC31PE immunotoxin. Data presented as median and box-and-whiskers plot with outliers. *POD* postoperative day (1–3), *M* morning sample, *E* evening sample. *P*-values < 0.05 considered statistically significant. *n.s.* nonsignificant

There was no difference between the groups for IFN-γ (Fig. 4). From the evening on the second postoperative day (E 2), IFN-γ increased in the MOC31PE group, while there was a time-dependent decrease in the CRS-HIPEC group. IP-10 increased time dependently in the MOC31PE group all three postoperative days, while in the CRS-HIPEC group, the increase plateaued from day 2 and onwards. There was no significant difference between the two groups.

The antiinflammatory cytokines IL-1RA and IL-10 displayed different time courses (Fig. 4). IL-1RA displayed a relatively flat curve with no particular time dependence the first three days postoperatively in either group. There was no significant difference between the two groups. IL-10 showed a time-dependent decrease the first 3 days postoperatively in both the MOC31PE and CRS-HIPEC group. The values were significantly higher in the MOC31PE than CRS-HIPEC group.

### IL-2, IL-4, IL-9, and IL-13

**Fig. 5:**
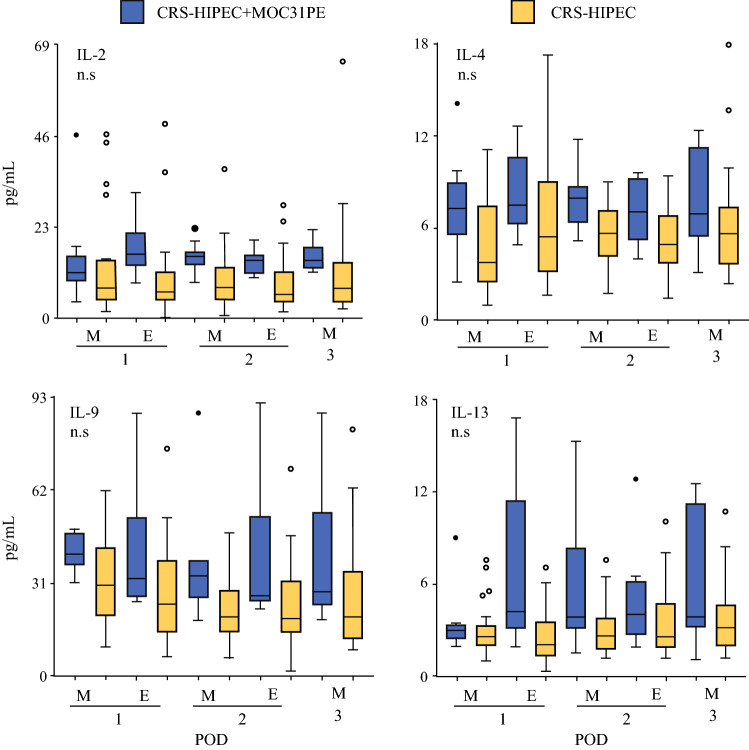
IL-2, IL-4, IL-9, and IL-13 in peritoneal fluid. Analysis of cytokines IL-2, IL-4, IL-9, and IL-13 in peritoneal fluid samples on the first three postoperative days after CRS-HIPEC with (*n* = 12, blue boxes) or without (*n* = 26, yellow boxes) intraperitoneal instillation of MOC31PE immunotoxin. Data presented as median and box-and-whiskers plot with outliers. *POD* postoperative day (1–3), *M* morning sample, *E* evening sample. *P*-values < 0.05 considered statistically significant. *n.s.* nonsignificant

The IL-2 and IL-13 levels in the MOC31PE group increased compared with the CRS-HIPEC group the first postoperative day, then plateaued (Fig. 5). No major time-dependent changes occurred in the CRS-HIPEC group. No major time-dependent change for IL-4 and IL-9 was detected in either group. There was no significant difference between the two groups for any of these four cytokines.

## Discussion

We identified a highly compartmentalized intraperitoneal inflammatory response that was clearly more pronounced in the MOC31PE group by analyzing local and systemic cytokine responses in patients undergoing CRS-HIPEC with or without additional MOC31PE treatment.

An important observation was that, although the patients were exposed to major surgery, the systemic inflammatory response was relatively modest while the local intraperitoneal response was substantial. Previously, systemic levels of a small number of cytokines have been analyzed in the setting of major surgery, typically to compare open versus minimally invasive surgery.^20^^,^^21^ In our study, the systemic response in serum induced by CRS-HIPEC with or without addition of MOC31PE was characterized by modest elevations in some proinflammatory cytokines, while for most cytokines, the results were similar to plasma levels in a reference population.^22^ The most pronounced elevation was detected for IL-6, which was 22 times higher than normal plasma levels, while other proinflammatory cytokines or chemokines, for instance IL-1β, IL-8, or IP-10, were detected at levels similar to the reference population. Several cytokines with detectable levels in a normal population were below the detection limit in serum in our study, e.g., the antiinflammatory IL-10 and the growth factor G-CSF. Interestingly, postoperative systemic cytokine levels were generally lower than levels for the same cytokines found in other studies after major surgery.^23^ This could be related to differences in sample composition, handling, and assay type, but it is also possible that mitomycin C (MMC)-based HIPEC could influence the production and release of cytokines compared with studies where patients had surgery only.

In contrast to the systemic response, a broad local inflammatory response was observed in samples collected from the peritoneal cavity after CRS-HIPEC. Compartmentalized inflammation is known from infection studies and is thought to be a defense mechanism to avoid potentially harmful systemic inflammation such as sepsis.^24^^,^^25^ Three main response patterns were identified, with the most prominent being an immediate substantial increase after surgery with a subsequent time-dependent gradual decrease. This pattern was typically seen for the proinflammatory cytokines IL-6, IL-1β, and IFN-γ, chemokines IL-8 and MCP-1, and growth factors G-CSF and FGF. Interestingly, the antiinflammatory cytokine IL-10 exhibited the same pattern, balancing the inflammatory response. The second main response pattern exhibited a minor initial increase followed by a flat time course, typically seen for TNF, MIP-1α, VEGF, and IL1RA. A third response pattern was observed for chemokine IP-10 only, being characterized by a gradual time-dependent increase.

Little is known about the initial local cytokine response after major abdominal surgery. A few studies have previously measured one to three cytokines in peritoneal fluid samples after abdominal surgery, typically analyzing one or more of the proinflammatory cytokines IL-6, TNF, and IL-1β and/or the chemokine IL-8.^23^^,^^26^ Individual cytokine levels varied greatly in these studies (for IL-6 between 16,000 and 139,000 pg/mL), and were generally higher than those measured in the present study. The absolute values are not necessarily directly comparable between studies because of differences in measurement method and sample handling, and as for the serum values, MMC-based HIPEC might influence release of cytokines from immune cells.

Under normal circumstances, there is very little fluid in the peritoneal cavity, and the baseline values of these 27 cytokines in peritoneal fluid are not known, making it challenging to interpret the magnitude of the inflammatory response; a relevant comparison could therefore be with systemic levels obtained in conditions with known extensive inflammatory responses. In a study of hospitalized, immunocompetent septic patients with confirmed bacteremia, cytokine plasma levels were measured by the same methodology as applied in the present study.^27^ The peak levels measured in peritoneal fluid after CRS-HIPEC were all notably higher, up to 700-fold for one of the cytokines, than the plasma levels of the septic patients. The biological significance of these high levels is unclear, but as we discuss below, many of these cytokines are involved in immunological responses that may be of relevance to cancer cell elimination.

When MOC31PE binds to EpCAM on the surface of cancer cells, it is internalized, upon which PE is cleaved off to cause inhibition of protein synthesis and induction of apoptosis.^6^^,^^28^ In addition to direct cell killing, we recently demonstrated that MOC31PE-induced cell death causes immune activation, measurable as a Th1 cytokine response, after intravenous administration to patients with end-stage metastatic CRC.^8^ In general, MOC31PE significantly enhanced a majority of local inflammatory/cytokine responses compared with CRS-HIPEC alone. Interestingly, increased levels of innate proinflammatory cytokines, such as IL-6, IL-1β, and TNF, as well as a time-dependent increase of the strong T-cell stimulator IFN-γ are all associated with ICD,^29^ and were all included in the broad cytokine response in the MOC31PE group. For IFN-γ, a time-dependent increase was observed in the MOC31PE group, in contrast to the time-dependent decrease seen in the CRS-HIPEC group. The increase coincided with a time-dependent increase in IP-10 in the MOC31PE group. The increases in IFN-γ and IP-10 were nonsignificant compared with the CRS-HIPEC group, but interestingly, the increase did not culminate in the observed time period and one might thus speculate that they have biological importance for the MOC31PE group. IP-10 has a distinct response pattern depending on the cause of inflammation. In a study of liver transplantation, IP-10 increased significantly in grafts with rejection but not in grafts with ischemia.^30^ IP-10 binds to CXCR3 receptor, which is called a “double-edged sword” in tumor progression, dependent on a range of factors including tumor origin, cells affected, isoform, and microenvironment.^31^ Evidence from preclinical models indicates that this IFN-γ-induced chemokine is associated with antitumor effects, such as reduced angiogenesis through inhibition of endothelial cell growth,^31^ mitotic activity and proliferation on endothelial cells, cytotoxic lymphocytes, natural killer (NK) cells, macrophages, and cancer cells.^32^

The biological and clinical relevance of the broad local inflammatory response observed in the peritoneal cavity after CRS-HIPEC is unclear, and in particular, it is difficult to predict the role of the observed intensification of the response after addition of MOC31PE in a cancer biology context. Tumor cell release of cytokines after ICD triggered by direct interaction with MOC31PE seems unlikely to explain the dramatic response, since a low number of tumor cells are present in the peritoneal cavity after complete CRS, thus other effects seem to be responsible. The antibody itself can initiate effects through fragment crystallizable (Fc)–Fc receptor binding, and by PE binding to pattern-recognition receptors on innate immune cells such as monocytes/macrophages and granulocytes. Analysis of immune cells in tissue and peritoneal fluid before and after CRS-HIPEC would be a logical next experimental step.

The main limitations of this study are related to the descriptive nature of the data and a somewhat heterogeneous study population with a limited number of patients. Importantly, although there were no differences in PCI score or extent of peritonectomy procedures, the heterogeneous nature of peritoneal metastasis spread means that it is difficult to completely standardize the groups. Based on our initial assumption that the inflammatory response would be mainly related to the surgical trauma, PMP patients were included in the CRS-HIPEC group. There were no systematic differences in inflammatory responses when comparing results from the PMP versus PM-CRC patients. However, although we cannot exclude that heterogeneity may have influenced the results to some extent, the compartmentalized response remains very clear, and the magnitude of the changes in the MOC31PE group also seem convincing. Thus, although causality cannot be concluded, the results may form an important basis for future studies.

Although the ImmunoPeCa trial’s primary end-point was safety, long-term follow-up revealed an estimated 78% 3-year OS,^10^ and updated follow-up data show an estimated 5-year OS of 53% (unpublished), which is excellent in a PM-CRC context. In the context of the present study, it seems that the intensified local inflammatory response was certainly not detrimental to the long-term outcome, and might even be beneficial.

In conclusion, a highly compartmentalized inflammatory response was observed in the peritoneal cavity after CRS-HIPEC for PM-CRC, in contrast to a modest systemic response. These results clearly underline the importance of analyzing local inflammatory responses, since systemic responses were minute even after major abdominal surgery. The local response was substantially enhanced by peritoneal instillation of MOC31PE, although a randomized study design would be necessary to prove causality. As the clinical results after the ImmunoPeCa trial are promising, it is tempting to speculate that the enhanced inflammatory response induced by MOC31PE may have been beneficial for the long-term outcome after CRS-HIPEC in PM-CRC.

## Supplementary Information

Below is the link to the electronic supplementary material.

Supplementary file 1 (DOCX 17 KB)

Supplementary file 2 (PDF 171 KB)

Supplementary file 3 (PDF 130 KB)

Supplementary file 4 (PDF 448 KB)

Supplementary file 5 (PDF 450 KB)

Supplementary file 6 (PDF 367 KB)

Supplementary file 7 (PDF 449 KB)

Supplementary file 8 (PDF 370 KB)
