# Supplementary material for: Increased Local Inflammatory Response to MOC31PE Immunotoxin After Cytoreductive Surgery and Hyperthermic Intraperitoneal Chemotherapy
Source: Ann Surg Oncol. 2021 May 21;28(9):5252–62. doi: 10.1245/s10434-021-10022-0 (PMC8349350; doi:10.1245/s10434-021-10022-0)
Supplement: Supplementary file 1 — (DOCX 17 KB) [file 10434_2021_10022_MOESM1_ESM.docx]

**Supplemental figure legends**

**Figure S1.** *Total protein amount in peritoneal fluid and serum cytokine response to intraperitoneal (IP) administered MOC31PE immunotoxin in serum postoperatively after CRS-HIPEC.*

Peritoneal fluid (PF) and serum was processed before and after operation from n = 12 patients operated with CRS-HIPEC and receiving the MOC31PE immunotoxin IP and from n = 26 patients operated with CRS-HIPEC. Total protein amount in the samples measured the first three postoperative days after CRS-HIPEC compared to CRS-HIPEC only are shown. Data are presented as median and box-and-whiskers plot with outliers. Grey boxes = CRS-HIPEC+MOC31PE. White boxes = CRS-HIPEC. M = morning sample. E = evening sample. Scr = screening before operation, POD = postoperative day (1 to 3). *P*-values <0.05 were considered statistically significant. n.s. = non-significant.

**Figure S2.** *Bio-Plex Human Cytokine 27-Plex Panel standard curve validation.*

The standard curve with the kit’s standard diluent was compared to standard curves with the standard diluent added 0.5 % bovine serum albumin (BSA) or 25 % Voluven (containing 60 mg/mL hydroxyethyl starch). Blue curves = standard diluent, red curves = standard diluent with 0.5 % BSA, green curves = standard diluent with 25 % Voluven.

**Figure S3.** *Proinflammatory cytokine response to intraperitoneal (IP) administered MOC31PE immunotoxin in serum postoperatively after CRS-HIPEC.*

Serum was processed before and after operation from n = 12 patients operated with CRS-HIPEC and receiving the MOC31PE immunotoxin IP and from n = 26 patients operated with CRS-HIPEC. The effects of MOC31PE on the proinflammatory cytokines IL-1β, IL-6 and TNF the first three postoperative days after CRS-HIPEC compared to CRS-HIPEC only are shown. Data are presented as median and box-and-whiskers plot with outliers. Scr = screening before operation, POD = postoperative day (1 to 3). Grey boxes = CRS-HIPEC+MOC31PE. White boxes = CRS-HIPEC.  *P*-values <0.05 were considered statistically significant, n.s. = non-significant.

**Figure S4.** *Chemokine response to intraperitoneal administered (IP) MOC31PE immunotoxin in serum postoperatively after CRS-HIPEC.*

Serum was processed before and after operation from n = 12 patients operated with CRS-HIPEC and receiving the MOC31PE immunotoxin IP and from n = 26 patients operated with CRS-HIPEC. The effects of MOC31PE on the chemokines IL-8, MCP-1, MIP-1α and MIP-1β the first three postoperative days after CRS-HIPEC compared to CRS-HIPEC only are shown. Data are presented as median and box-and-whiskers plot with outliers. Scr = screening before operation, POD = postoperative day (1 to 3). Grey boxes = CRS-HIPEC+MOC31PE. White boxes = CRS-HIPEC. *P*-values <0.05 were considered statistically significant. , n.s. = non-significant.

**Figure S5.** *Growth factor response to intraperitoneal administered (IP) MOC31PE immunotoxin in serum postoperatively after CRS-HIPEC.*

Serum was processed before and after operation from n = 12 patients operated with CRS-HIPEC and receiving the MOC31PE immunotoxin IP and from n = 26 patients operated with CRS-HIPEC. The effects of MOC31PE on the growth factors IL-7 and FGF the first three postoperative days after CRS-HIPEC compared to CRS-HIPEC only are shown. Data are presented as median and box-and-whiskers plot with outliers. The Mann-Whitney *U* test was used to determine statistical significance between the two groups at each time point. Scr = screening before operation, POD = postoperative day (1 to 3). Grey boxes = CRS-HIPEC+MOC31PE. White boxes = CRS-HIPEC.  *P*-values <0.05 were considered statistically significant.

**Figure S6.** *IFN-γ and IP-10, IL-1RA and IL-10 response to intraperitoneal administered (IP) MOC31PE immunotoxin in serum postoperatively after CRS-HIPEC.*

Serum was processed before and after operation from n = 12 patients operated with CRS-HIPEC and receiving the MOC31PE immunotoxin IP and n = 26 patients operated with CRS-HIPEC. The effects of MOC31PE on IFN-γ and IP-10, the anti-inflammatory cytokine IL-1RA the first three postoperative days after CRS-HIPEC compared to CRS-HIPEC only are shown. Data are presented as median and box-and-whiskers plot with outliers. The Mann-Whitney *U* test was used to determine statistical significance between the two groups at each time point. Scr = screening before operation, POD = postoperative day (1 to 3). Grey boxes = CRS-HIPEC+MOC31PE. White boxes = CRS-HIPEC. *P*-values <0.05 were considered statistically significant. n.s. = non-significant.

**Figure S7.** *IL-2, IL-4 and IL-9 response to intraperitoneal administered (IP) MOC31PE immunotoxin in serum postoperatively after CRS-HIPEC.*

Serum was processed before and after operation from n = 12 patients operated with CRS-HIPEC and receiving the MOC31PE immunotoxin IP and n = 26 patients operated with CRS-HIPEC. The effects of MOC31PE on the cytokines IL-2, IL-4 and IL-9 the first three postoperative days after CRS-HIPEC compared to CRS-HIPEC only are shown. Data are presented as median and box-and-whiskers plot with outliers. Scr = screening before operation, POD = postoperative day (1 to 3). Grey boxes = CRS-HIPEC+MOC31PE. White boxes = CRS-HIPEC. *P*-values <0.05 were considered statistically significant. , n. s. = non-significant.
