# Supplementary figures and images for: Increased Local Inflammatory Response to MOC31PE Immunotoxin After Cytoreductive Surgery and Hyperthermic Intraperitoneal Chemotherapy
Source: Ann Surg Oncol. 2021 May 21;28(9):5252–62. doi: 10.1245/s10434-021-10022-0 (PMC8349350; doi:10.1245/s10434-021-10022-0)

■ CRS-HIPEC+MOC31PE    □ CRS-HIPEC

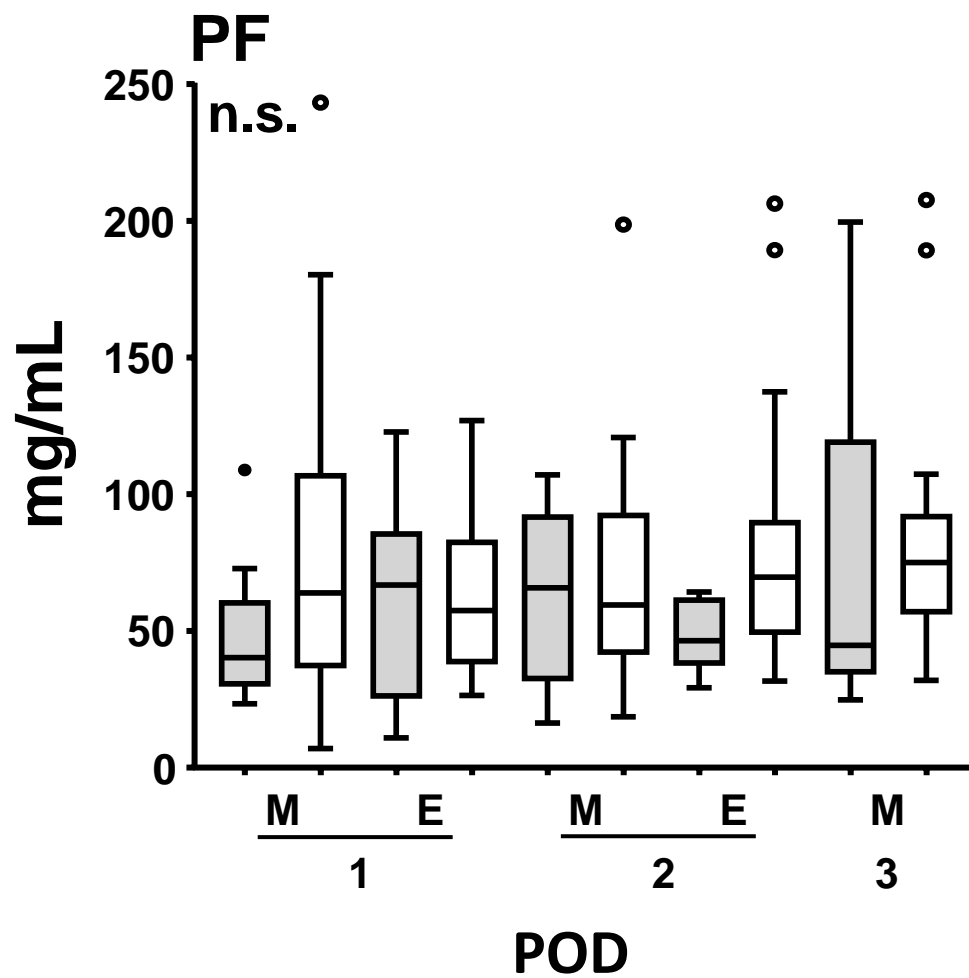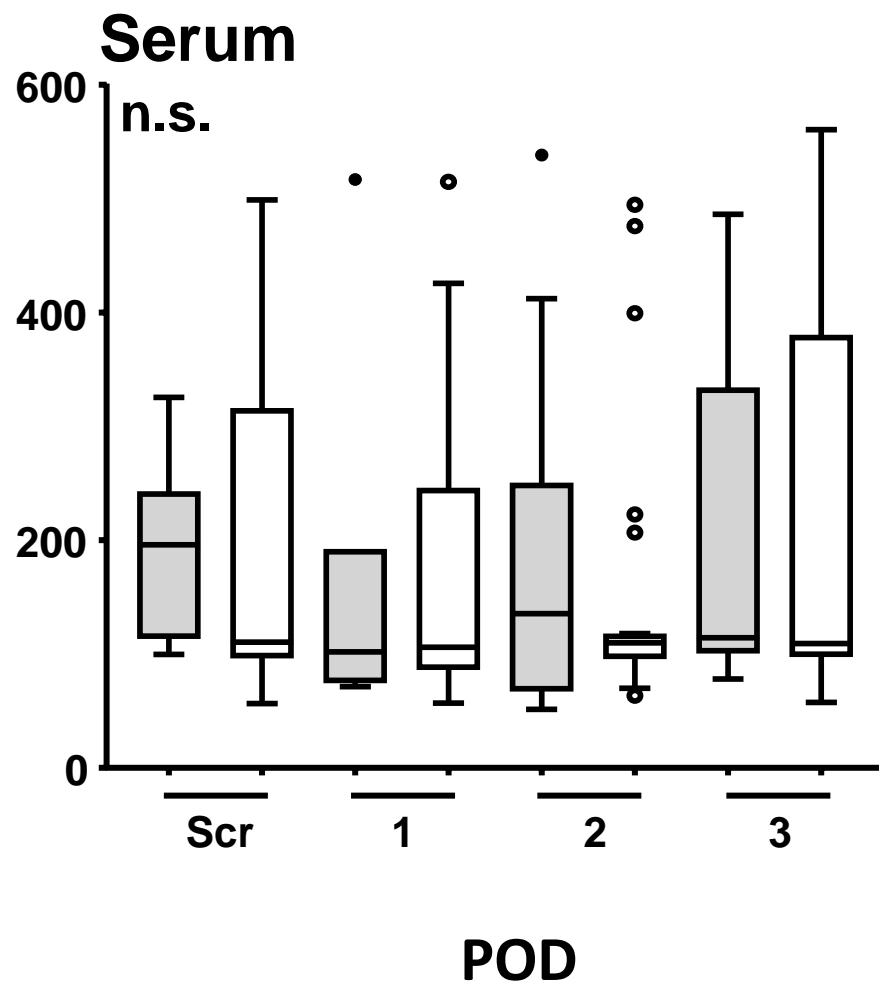

Supplement: Supplementary file 2 — (PDF 171 KB) [file 10434_2021_10022_MOESM2_ESM.pdf]

■ CRS-HIPEC+MOC31PE    □ CRS-HIPEC

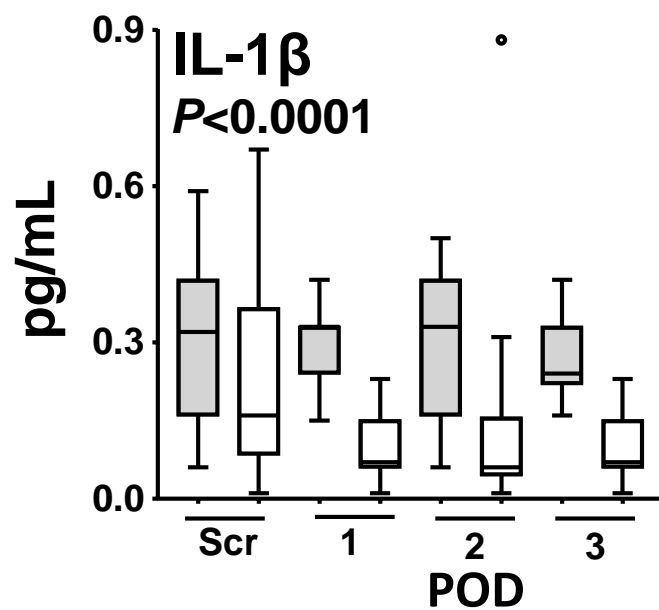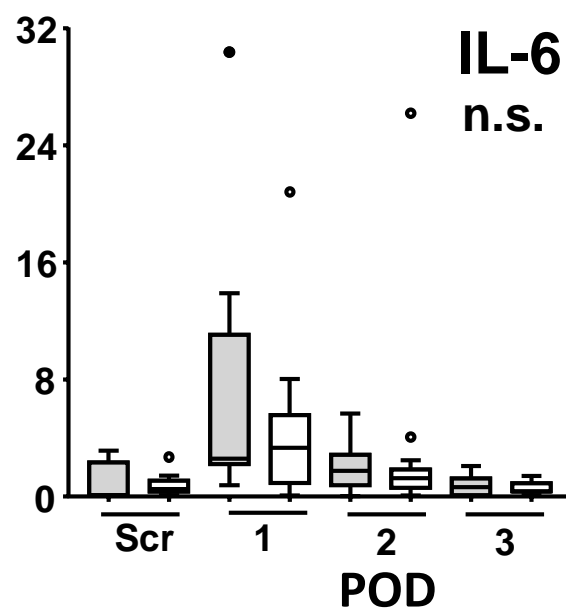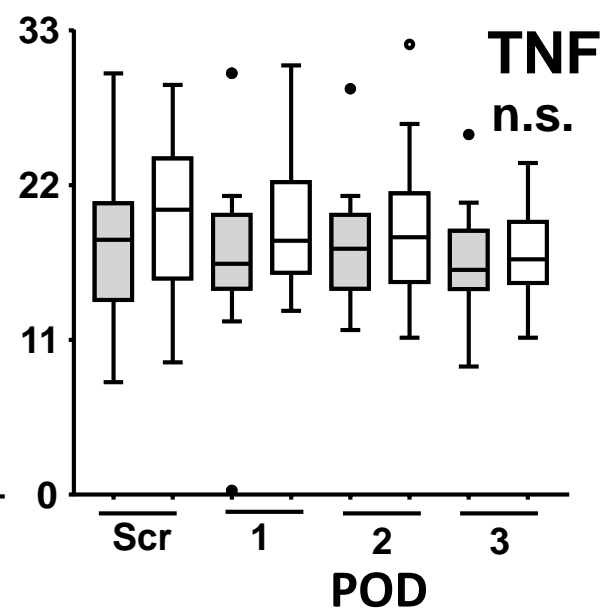

Supplement: Supplementary file 4 — (PDF 448 KB) [file 10434_2021_10022_MOESM4_ESM.pdf]

■ CRS-HIPEC+MOC31PE    □ CRS-HIPEC

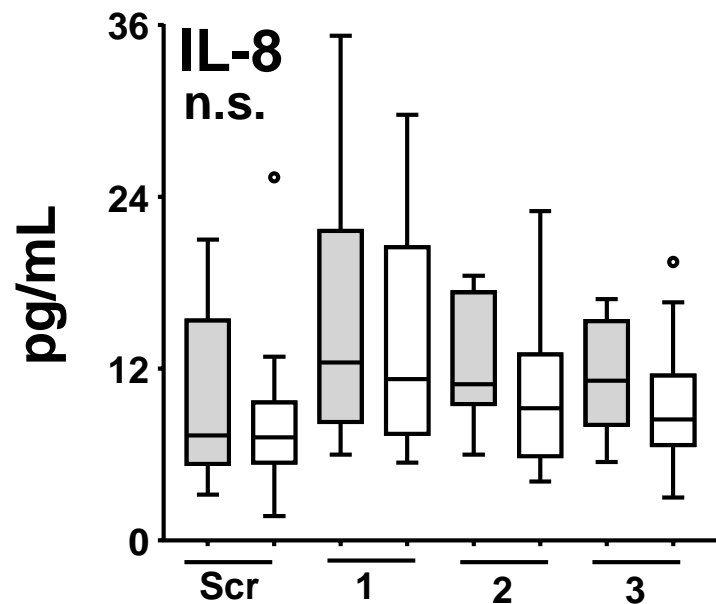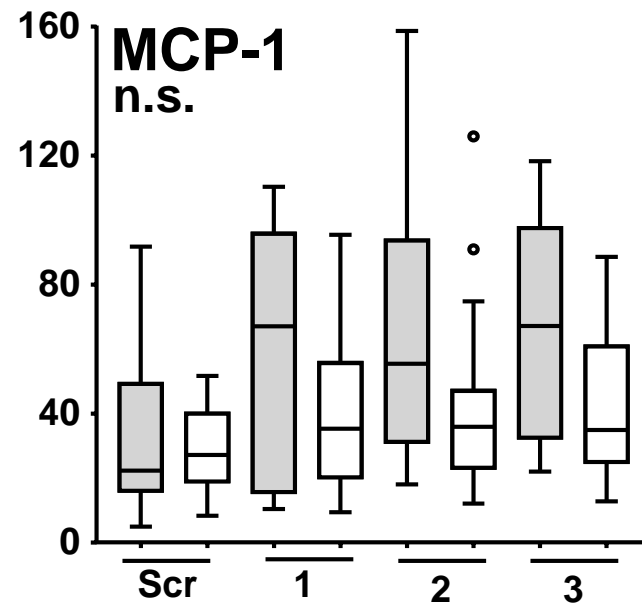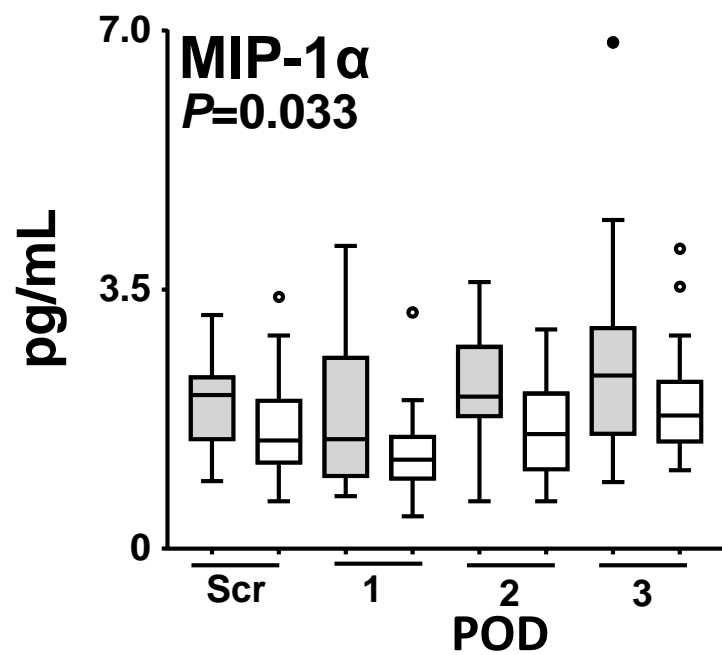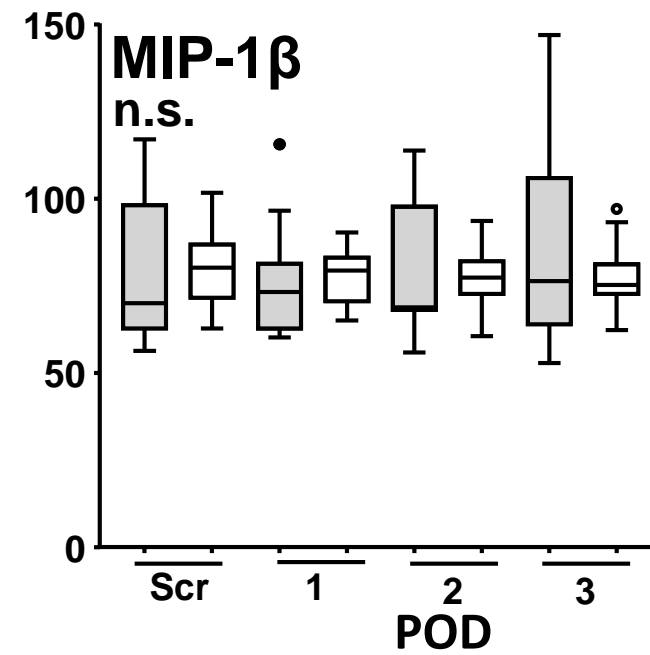

Supplement: Supplementary file 5 — (PDF 450 KB) [file 10434_2021_10022_MOESM5_ESM.pdf]

■ CRS-HIPEC+MOC31PE    □ CRS-HIPEC

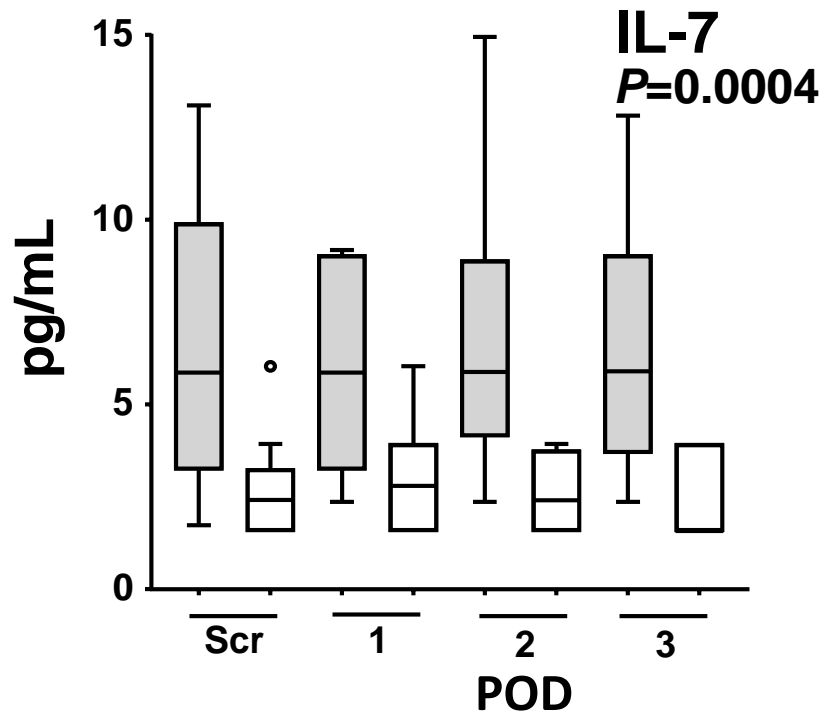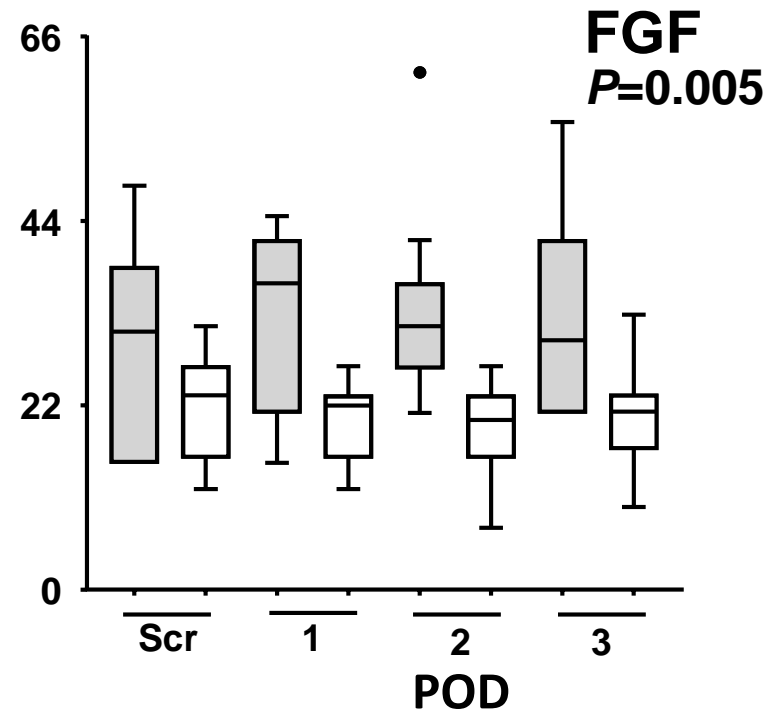

Supplement: Supplementary file 6 — (PDF 367 KB) [file 10434_2021_10022_MOESM6_ESM.pdf]

■ CRS-HIPEC+MOC31PE    □ CRS-HIPEC

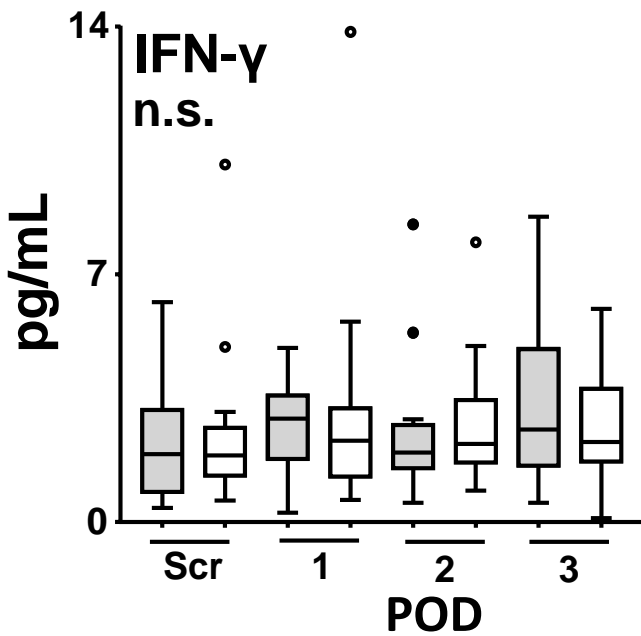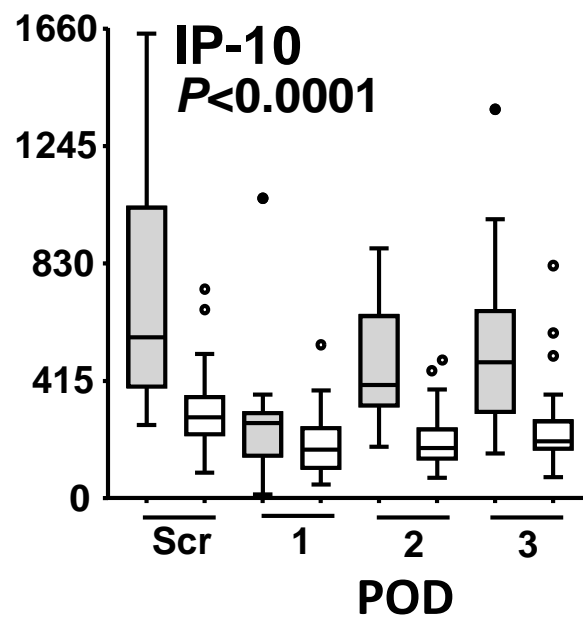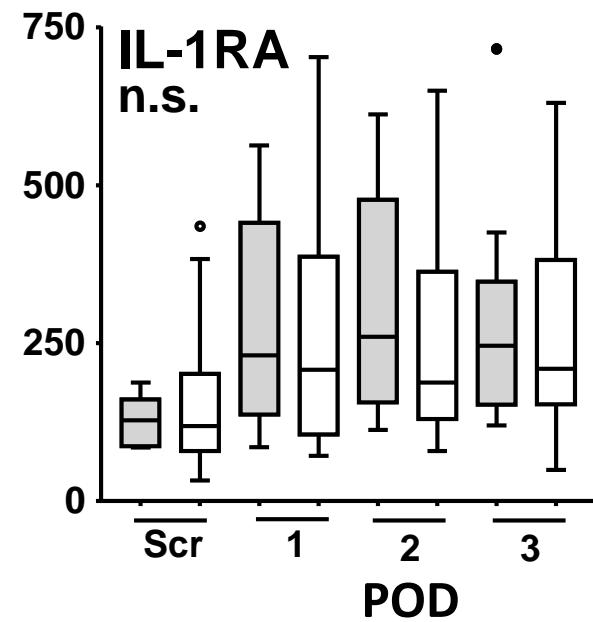

Supplement: Supplementary file 7 — (PDF 449 KB) [file 10434_2021_10022_MOESM7_ESM.pdf]

■ CRS-HIPEC+MOC31PE    □ CRS-HIPEC

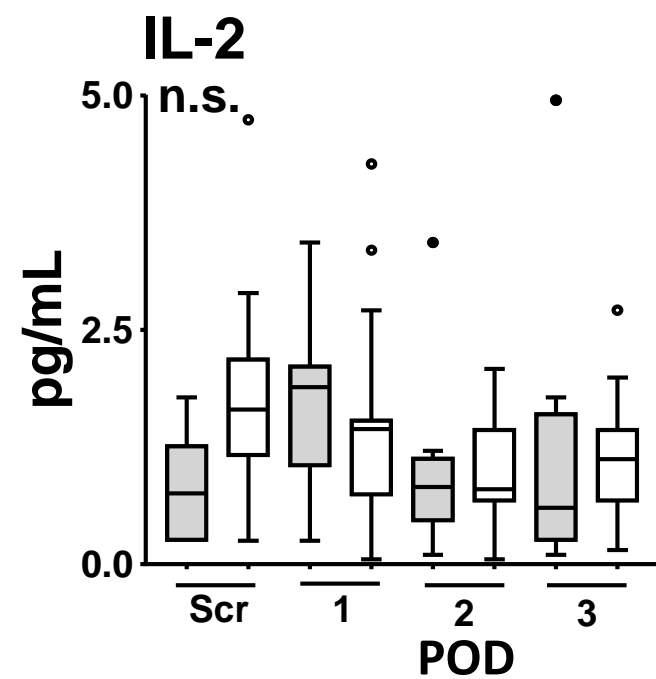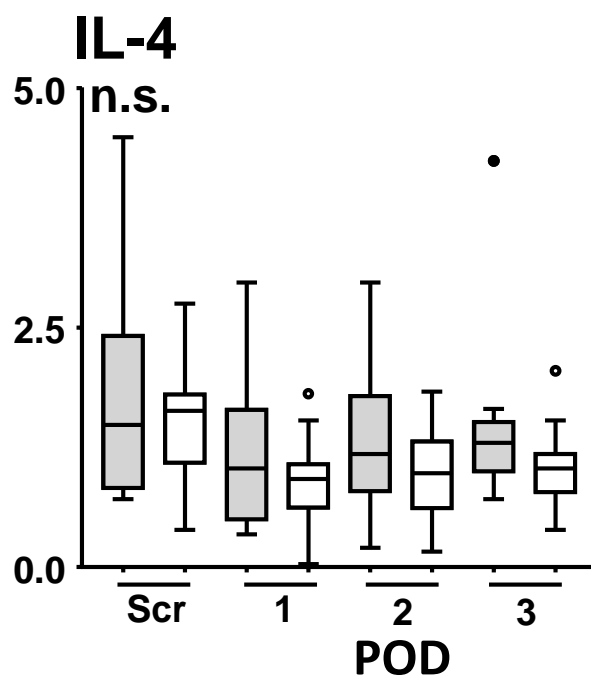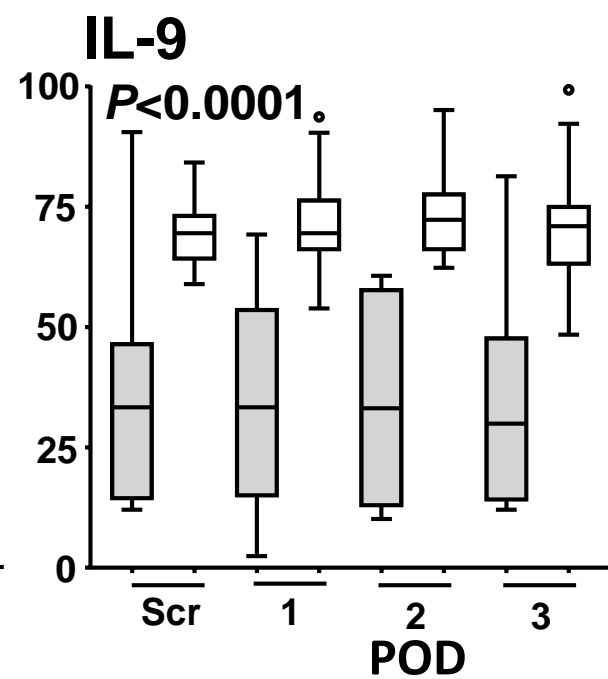

Supplement: Supplementary file 8 — (PDF 370 KB) [file 10434_2021_10022_MOESM8_ESM.pdf]
