# Supplementary material for: Increased Local Inflammatory Response to MOC31PE Immunotoxin After Cytoreductive Surgery and Hyperthermic Intraperitoneal Chemotherapy
Source: Ann Surg Oncol. 2021 May 21;28(9):5252–62. doi: 10.1245/s10434-021-10022-0 (PMC8349350; doi:10.1245/s10434-021-10022-0)
Supplement: Supplementary file 3 — (PDF 130 KB) [file 10434_2021_10022_MOESM3_ESM.pdf]

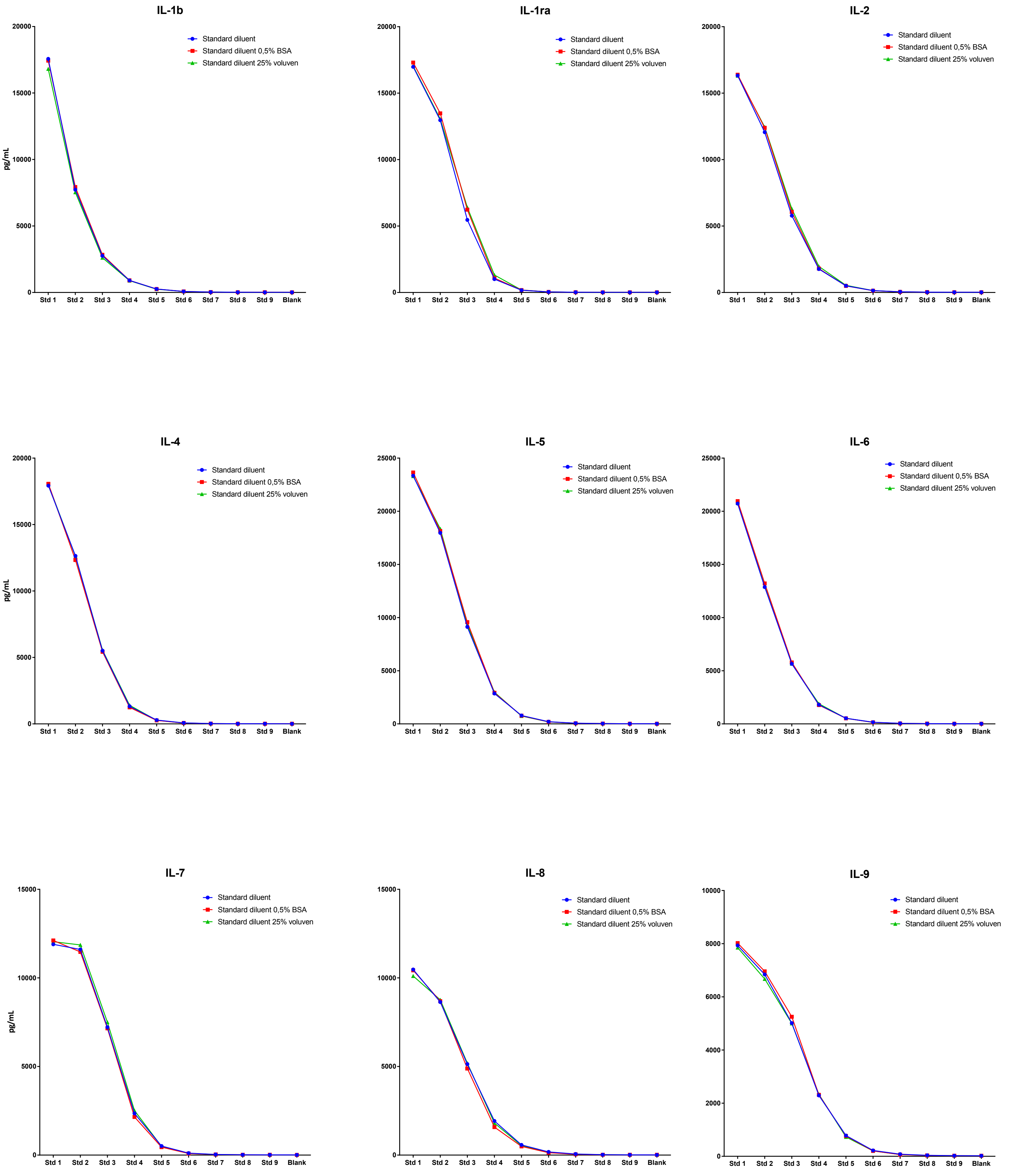
IL-1b
IL-1ra
IL-2
IL-4
IL-5
IL-6
IL-7
IL-8
IL-9
pg/mL
Standard diluent
Standard diluent 0,5% BSA
Standard diluent 25% voluven
Std 1
Std 2
Std 3
Std 4
Std 5
Std 6
Std 7
Std 8
Std 9
Blank
0
2000
4000
5000
6000
8000
10000
15000
20000
25000

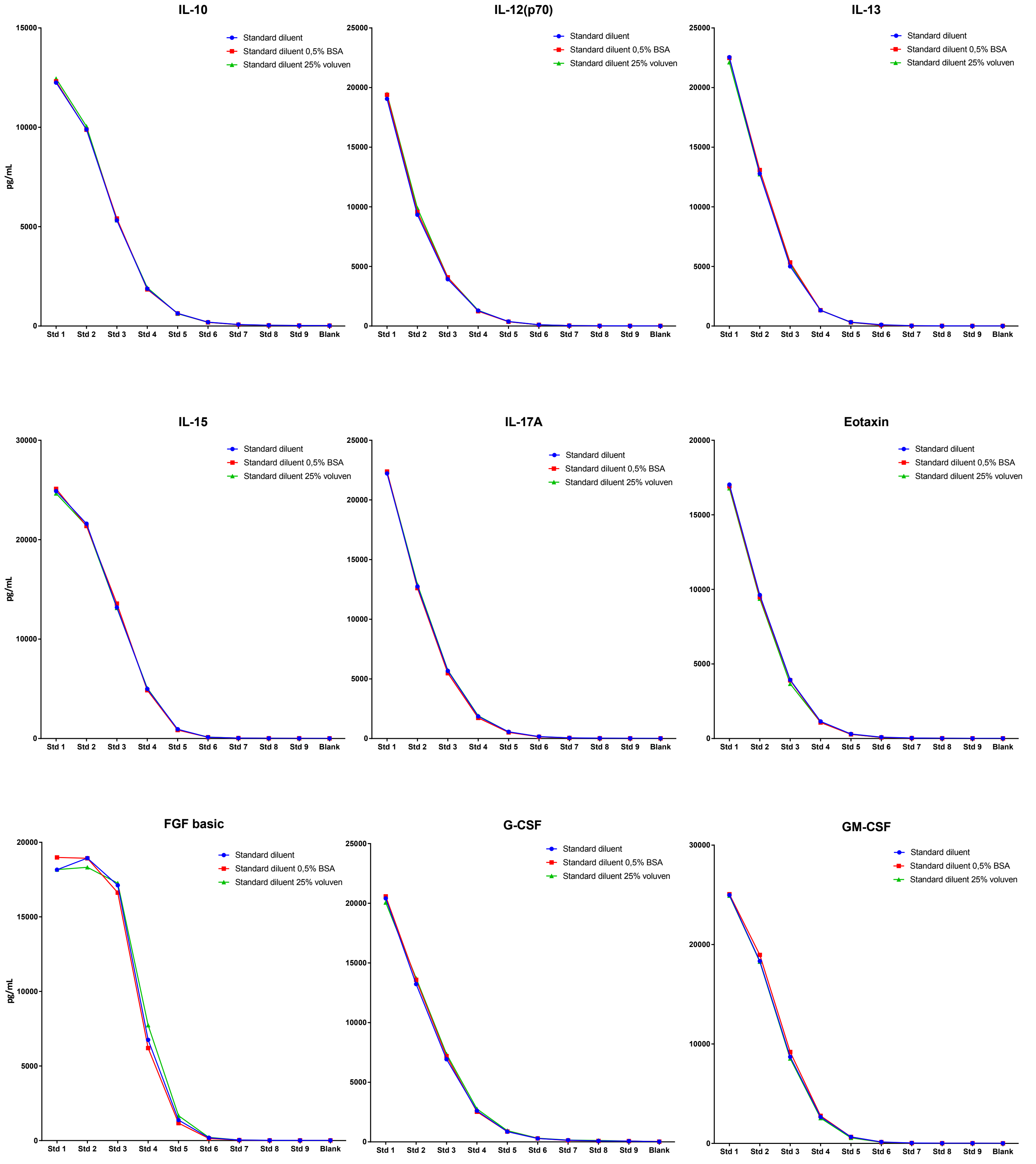
IL-10
IL-12(p70)
IL-13
IL-15
IL-17A
Eotaxin
FGF basic
G-CSF
GM-CSF
pg/mL
Standard diluent
Standard diluent 0,5% BSA
Standard diluent 25% voluven
Std 1
Std 2
Std 3
Std 4
Std 5
Std 6
Std 7
Std 8
Std 9
Blank
0
5000
10000
15000
20000
25000
30000

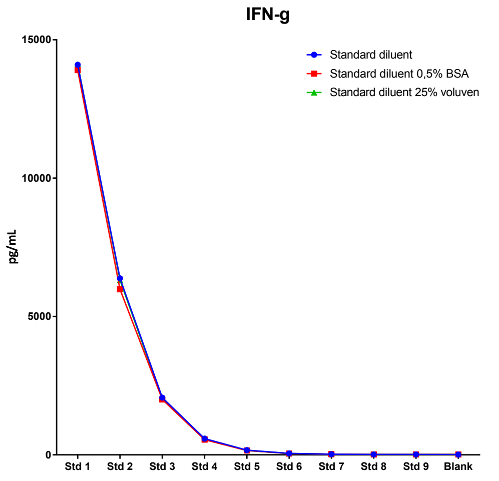
IFN-g
pg/mL
15000
10000
5000
0
Std 1 Std 2 Std 3 Std 4 Std 5 Std 6 Std 7 Std 8 Std 9 Blank
Standard diluent
Standard diluent 0,5% BSA
Standard diluent 25% voluven

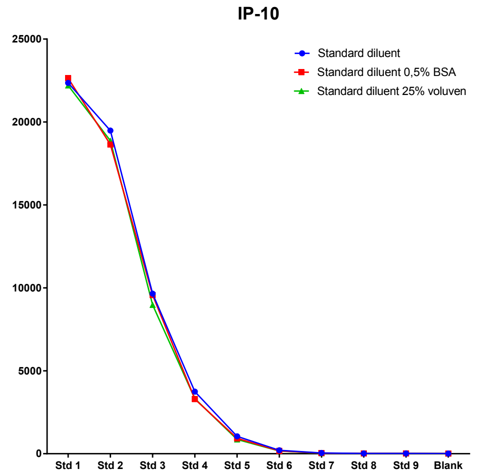
IP-10
25000
20000
15000
10000
5000
0
Std 1 Std 2 Std 3 Std 4 Std 5 Std 6 Std 7 Std 8 Std 9 Blank
Standard diluent
Standard diluent 0,5% BSA
Standard diluent 25% voluven

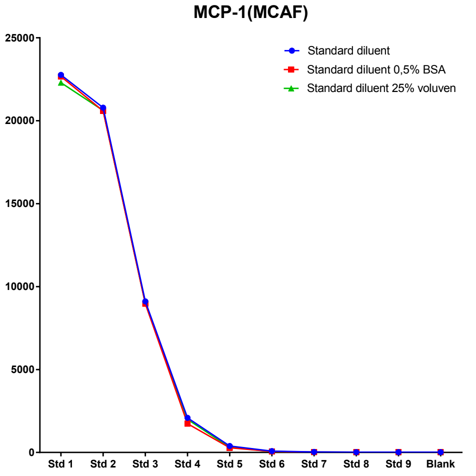
MCP-1(MCAF)
25000
20000
15000
10000
5000
0
Std 1 Std 2 Std 3 Std 4 Std 5 Std 6 Std 7 Std 8 Std 9 Blank
Standard diluent
Standard diluent 0,5% BSA
Standard diluent 25% voluven

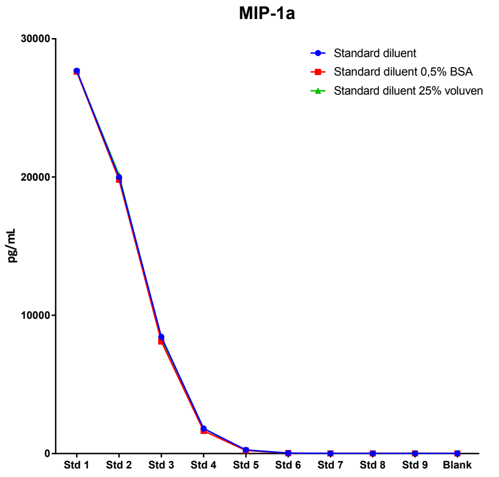
MIP-1a
pg/mL
30000
20000
10000
0
Std 1 Std 2 Std 3 Std 4 Std 5 Std 6 Std 7 Std 8 Std 9 Blank
Standard diluent
Standard diluent 0,5% BSA
Standard diluent 25% voluven

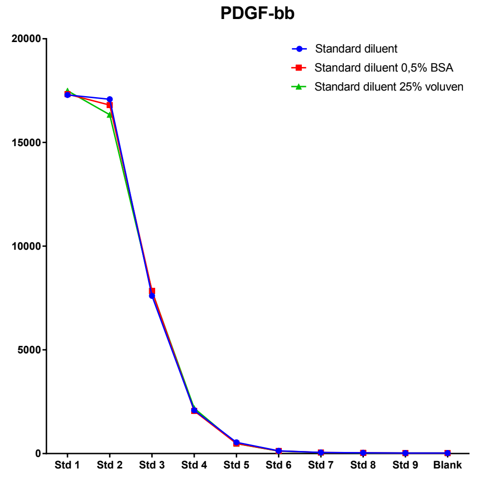
PDGF-bb
20000
15000
10000
5000
0
Std 1 Std 2 Std 3 Std 4 Std 5 Std 6 Std 7 Std 8 Std 9 Blank
Standard diluent
Standard diluent 0,5% BSA
Standard diluent 25% voluven

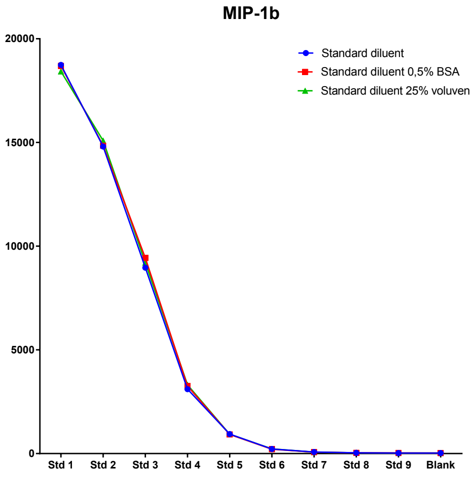
MIP-1b
20000
15000
10000
5000
0
Std 1 Std 2 Std 3 Std 4 Std 5 Std 6 Std 7 Std 8 Std 9 Blank
Standard diluent
Standard diluent 0,5% BSA
Standard diluent 25% voluven

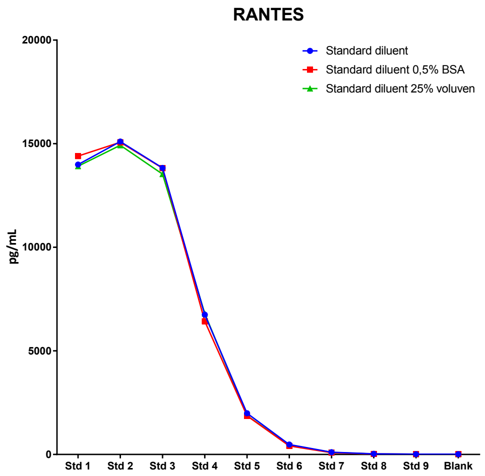
RANTES
pg/mL
20000
15000
10000
5000
0
Std 1 Std 2 Std 3 Std 4 Std 5 Std 6 Std 7 Std 8 Std 9 Blank
Standard diluent
Standard diluent 0,5% BSA
Standard diluent 25% voluven

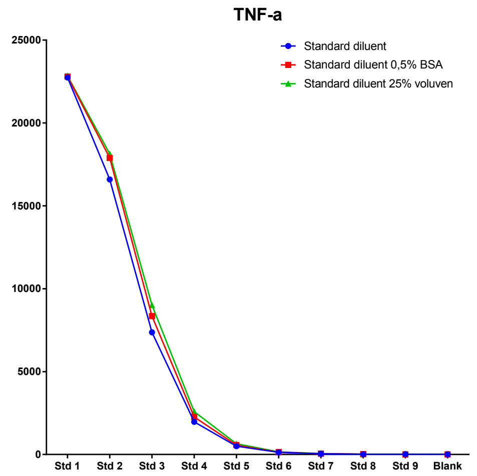
TNF-a
25000
20000
15000
10000
5000
0
Std 1 Std 2 Std 3 Std 4 Std 5 Std 6 Std 7 Std 8 Std 9 Blank
Standard diluent
Standard diluent 0,5% BSA
Standard diluent 25% voluven

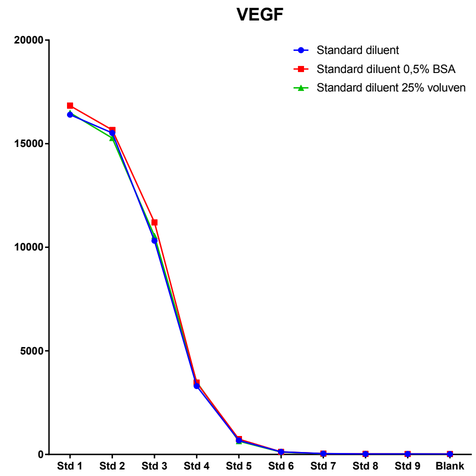
VEGF
20000
15000
10000
5000
0
Std 1 Std 2 Std 3 Std 4 Std 5 Std 6 Std 7 Std 8 Std 9 Blank
Standard diluent
Standard diluent 0,5% BSA
Standard diluent 25% voluven
